# Transcriptomic Profiling Reveals the Seed Aging Process in *Elymus sibiricus*, a Dominant Alpine Grass

**DOI:** 10.3390/plants15091328

**Published:** 2026-04-27

**Authors:** Ming Sun, Li Wang, Xinchao Sun, Jiajun Yan, Wenlong Gou, Jing Liu, Chanjuan Wu, Yilin He, Guo Yue, Dongbin Li, Rongxia Wang, Xiong Lei, Shiqie Bai

**Affiliations:** 1 College of Life Sciences and Agri-Forestry, Southwest University of Science and Technology, Mianyang 621010, China; sunming@swust.edu.cn (M.S.); wangli4197@163.com (L.W.); zhifengcao510@126.com (J.Y.); gwl-395124@swust.edu.cn (W.G.); liujing2024@swust.edu.cn (J.L.); wuchanjuan@swust.edu.cn (C.W.); 19218239799@163.com (Y.H.); yueguo3466@163.com (G.Y.); wangrongxia2022@163.com (R.W.); 2 College of Life Science and Technology, Gansu Agricultural University, Lanzhou 730070, China; 19968506064@163.com; 3 Sichuan Zhihechengrui Agriculture & Animal Husbandry Technology Co., Ltd., Chengdu 610306, China; 18283582828@163.com; 4 Sichuan Academy of Grassland Sciences, Chengdu 611731, China

**Keywords:** seed aging, *Elymus sibiricus*, transcriptome, WGCNA, transcription factor

## Abstract

Seed aging is a critical biological process that leads to progressive loss of seed vigor, thereby constraining germplasm conservation and agricultural productivity. To elucidate the molecular mechanisms underlying this process in grass species, we performed transcriptomic analyses to characterize regulatory networks underlying seed aging in *Elymus sibiricus*, a dominant forage species on the Qinghai–Tibet Plateau. Seeds were subjected to artificial accelerated aging (45 °C, 80% relative humidity, 1–6 days), followed by physiological evaluation and RNA sequencing. Seed vigor and germination percentage declined markedly with aging, accompanied by extensive transcriptional reprogramming. Integrative analyses identified pyruvate metabolism, MAPK signaling, and peroxisome function as key processes associated with vigor loss during late-stage aging. WGCNA further revealed that genes encoding heat shock proteins and glutathione metabolism-related enzymes were co-localized within the same module, suggesting a possible synergistic role in preserving seed viability during aging. In addition, WRKY24, ARF9, and ARF19 were identified as candidate hub transcription factors. WRKY24 may contribute to aging by modulating antioxidant defense-related genes (e.g., *TRX1* and *NRPC1*), while ARF9 and ARF19 may regulate ROS homeostasis through predicted downstream targets, including *FQR1*, *PER2*, *MAO1B*, *ANN5*, and *MT2B*. Together, these findings support a hypothetical regulatory model in which WRKY and ARF transcription factors coordinate redox homeostasis and hormone signaling to regulate seed longevity in *E. sibiricus*. This study provides a systems-level framework for understanding seed aging in perennial grasses and identifies potential genetic targets for improving seed storability, with implications for germplasm conservation and alpine grassland sustainability.

## 1. Introduction

*Elymus sibiricus*, a perennial, self-pollinating allotetraploid (StStHH) grass within the Poaceae family and the *Elymus* genus, serves as a representative model for this taxon [1]. Consequently, research on *E. sibiricus* provides valuable insights into the broader *Elymus* genus. The Qinghai–Tibet Plateau harbors extensive wild resources of *E. sibiricus*, whose germplasm is of considerable value for grassland restoration, livestock production, and the genetic improvement in related crops, including common wheat [2,3]. Globally, extensive germplasm resources of the *Elymus* genus have been collected, underscoring the importance of studies on genetic diversity and conservation for effective utilization and biodiversity preservation [4].

*E. sibiricus* produces caryopsis-type seeds, with the embryo and endosperm enclosed by persistent lemma and palea. At maturity, the seeds exhibit non-deep physiological dormancy, which can be effectively released by dry after-ripening, cold stratification, or GA_3_ treatment [5]. Under field conditions, germination and seedling emergence are often slow and uneven due to fluctuations in soil moisture and temperature. Under laboratory conditions (alternating temperatures of 25/15 °C), seeds collected 60 days after anthesis show a germination rate of only approximately 11% [5]. However, after one year of natural storage, the germination percentage can reach over 95%, followed by a gradual decline with prolonged storage [6]. These morphological and physiological characteristics provide a useful framework for investigating seed aging behavior and vigor dynamics.

Seed aging is an inevitable process during storage, leading to reduced germination and progressive loss of seed vigor, thereby compromising agricultural stability and ecosystem sustainability. This process is influenced by both environmental and genetic factors [7], and different germplasm resources often exhibit considerable heterogeneity in seed vigor traits, including aging tolerance, germination capacity, and dormancy levels, primarily governed by intrinsic genetic regulatory mechanisms [8]. However, natural seed aging is inherently slow and subject to environmental variability, which constrains mechanistic investigation. Accelerated aging methods, such as the accelerated aging test (AAT) and controlled deterioration test (CDT), have been standardized by the International Seed Testing Association (ISTA) and provide controlled, time-efficient surrogate systems that recapitulate key features of natural aging. Accordingly, AAT is widely employed across model and crop species, including *Arabidopsis thaliana* [8], rice (*Oryza sativa*) [9], and oat (*Avena sativa*) [10], facilitating the dissection of physiological and genetic mechanisms underlying seed deterioration.

The seed aging process triggers a cascade of detrimental physiological and molecular alterations, including DNA damage, loss of plasma membrane integrity, organellar disorganization, accumulation of toxic metabolites, dysregulation of hormone signaling, and impairment of antioxidant defense systems. In recent years, increasing evidence has identified and functionally validated numerous regulatory factors, including transcription factors and enzymes, that play crucial roles in seed aging. Genes associated with redox homeostasis, hormone signaling, and energy metabolism have been shown to act as key components of this regulatory network. Representative examples include antioxidant genes such as superoxide dismutase (*CuSOD*), ascorbate peroxidase (*APX*) [11], dehydroascorbate reductase (*DHAR*) [8], glutathione reductase (*GR*) [12], thioredoxin (*TRX*) [12], and peroxiredoxin 1A (*PER1A*) [9], as well as transcription factors and stress-responsive genes such as *bZIP23* [9], wall-associated kinase (*WAK16*) [13], small auxin-up RNA gene (*SAUR33*) [14], lipoxygenase (*LOX1*) [15], and respiratory burst oxidase homologs (*RBOHs*) [8]. In addition to redox regulation, hormone signaling plays an indispensable role in controlling seed aging and longevity. Phytohormones such as auxins, gibberellins, and abscisic acid (ABA) have been recognized as critical regulators of seed lifespan. Among these, ABA is a key hormone mediating the acquisition and maintenance of seed longevity, primarily through the action of abscisic acid insensitive 3 (*ABI3*) and abscisic acid insensitive 5 (*ABI5*) [16]. Crosstalk between auxin and ABA signaling pathways has also been implicated in the modulation of seed longevity; for instance, activation of the *TIR1/ABF–ARF* signaling cascade promotes *ABI3* expression, thereby influencing seed aging regulation. Moreover, hormone signaling can affect seed lifespan indirectly through the regulation of ROS homeostasis. In rice, *OsSAUR33* expression is markedly upregulated in aged seeds compared with unaged seeds during germination, and *OsSAUR33* disruption significantly reduces seed vigor under both natural storage and artificial aging conditions [14]. Despite these advances, knowledge regarding the molecular regulation of seed longevity in *Elymus* species remains limited. Given the ecological and agricultural importance of *E. sibiricus* and related taxa, further exploration of their transcriptional responses during seed aging is of particular significance. Characterizing the expression dynamics of longevity-associated genes and their interactions across redox, hormonal, and metabolic pathways will provide critical insights into seed vigor maintenance in this genus.

In the present study, we utilized the high-seed-yield cultivar *E. sibiricus* ‘Maiwa’, which is widely employed for grassland restoration and artificial pasture establishment on the Qinghai–Tibet Plateau. The recent release of a high-quality reference genome for *E. sibiricus* provides a crucial resource that enhances the accuracy and depth of multi-omics investigations. Here, we aimed to delineate the gene expression profiles associated with seed aging by integrating transcriptomic profiling with germination phenotypes. This work seeks to identify key regulatory genes underlying seed aging and to advance the mechanistic understanding of seed vigor in perennial grasses.

## 2. Results

### 2.1. Impact of Artificial Aging on Seed Germination

**Figure 1:**
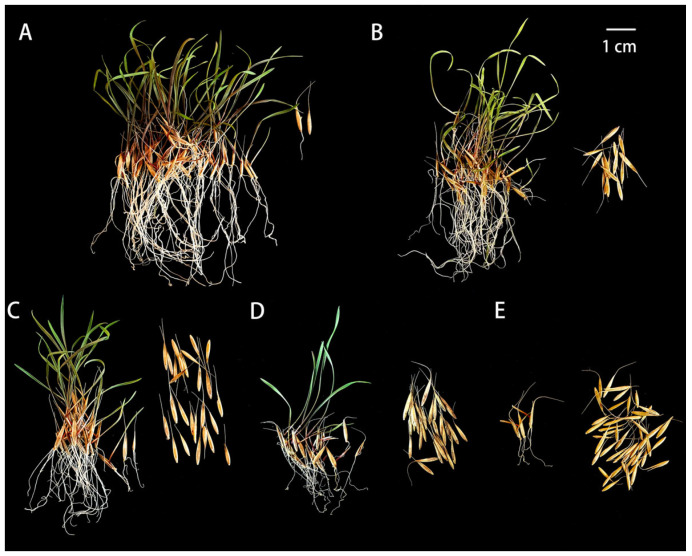
Phenotypes of *E. sibiricus* seeds during germination after different aging treatments. Seed germination phenotypes after 12 days of imbibition following aging treatments for 0 d (**A**), 1 d (**B**), 2 d (**C**), 4 d (**D**), and 6 d (**E**) at 45 °C and 80% relative humidity.

Before aging treatments, the control seeds (A0) exhibited high viability, with a germination percentage of 94% and a radicle emergence percentage of 96%. Both parameters declined progressively and nearly linearly with increasing aging duration (Figure 1). After one day of aging (A1), germination percentage and radicle emergence percentage decreased to 63% and 69%, respectively. The decline continued after two days (A2; 40% and 49%) and four days (A4; 10% and 35%). After six days (A6), no normal seedlings were observed (0%), and only 5% of seeds exhibited radicle emergence (Figure 1E).

**Figure 2:**
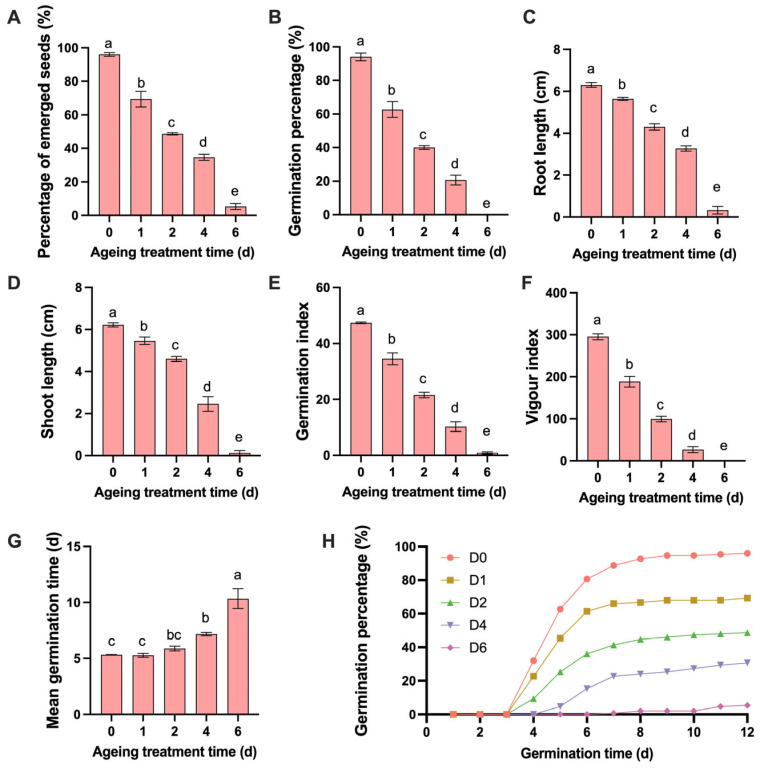
Effects of aging duration on seed germination and early seedling growth. (**A**) Radicle emergence percentage (EP). (**B**) Germination percentage (GP). (**C**) Root length (RL). (**D**) Shoot length (SL). (**E**) Germination index (GI). (**F**) Vigor index (VI). (**G**) Mean germination time (MGT). (**H**) Germination curve. Data are presented as means ± SEM (*n* = 3). Different lowercase letters indicate significant differences at *p* < 0.05 (Duncan’s multiple range test).

This decline in germination performance was accompanied by a marked reduction in vigor-related traits, including shoot length, root length, germination index, and vigor index (Figure 2). Aging also delayed germination kinetics, as indicated by an increase in mean germination time from 5.32 days (A0) to 10.33 days (A6) (Figure 2G). Together, these results confirm that artificial aging severely impairs seed vigor and germination efficiency in *E. sibiricus*. Most non-germinated aged seeds softened during imbibition, and the remained hard seeds did not respond to dormancy-breaking treatments, indicating that aging did not induce secondary dormancy.

### 2.2. Dynamic Alterations in the Transcriptomic Landscape in Response to Aging

**Figure 3:**
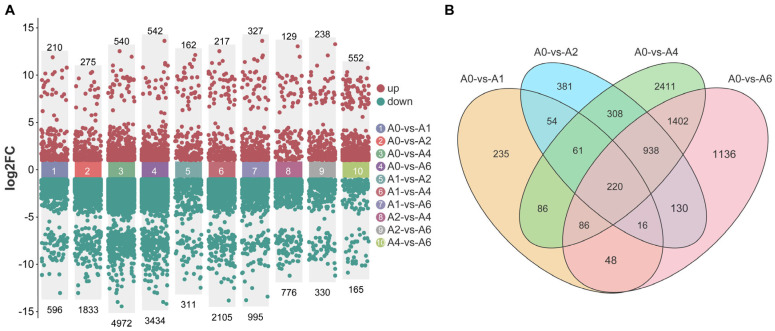
Effects of aging on seed gene expression profiles. (**A**) Statistics of DEGs identified from pairwise comparisons among the five sample groups. (**B**) Venn diagram showing overlap of DEGs between each aging treatment (A1, A2, A4, and A6) and the unaged control group (A0).

Transcriptome profiling revealed extensive transcriptional reprogramming during artificial aging. Principal component analysis (PCA) of all 15 samples showed clear clustering of biological replicates and progressive separation among aging stages (Figure S1). The number of differentially expressed genes (DEGs) increased with aging duration up to day 4 and then declined at day 6, likely reflecting the high proportion of non-viable cells. Compared with A0, 806, 2108, 5512, and 3976 DEGs were identified in A1, A2, A4, and A6, respectively. Downregulated genes predominated in all comparisons, peaking at 4972 in A4 vs. A0, while upregulated genes reached their maximum at 540 (A4) and 542 (A6) (Figure 3A).

Across all aging stages (A1–A6), a total of 220 DEGs were identified as shared, underscoring their sustained roles in the seed aging process. In contrast, substantial numbers of stage-specific DEGs emerged—235 in A1, 381 in A2, 2411 in A4, and 1136 in A6—highlighting distinct transcriptional profiles associated with different physiological transitions, particularly the pronounced transcriptomic collapse observed at the late-aging stage A6 (Figure 3B).

### 2.3. Metabolic Pathway and Regulatory Network Analysis of DEGs

**Figure 4:**
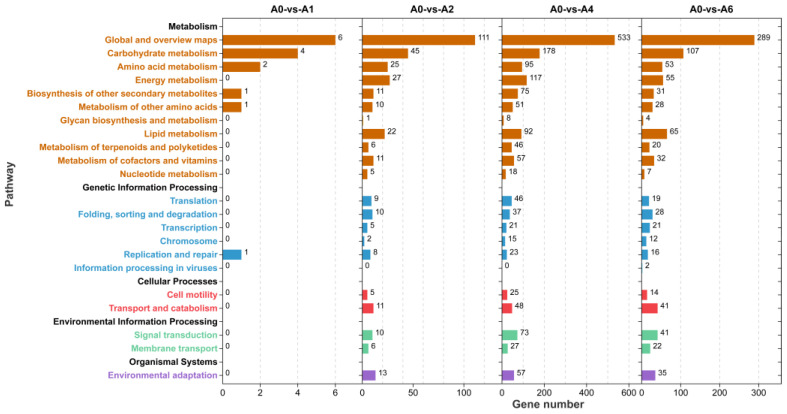
Overview of KEGG pathway enrichment of DEGs across aging stages in *E. sibiricus* seeds.

The KEGG pathway distribution of DEGs exhibited a pattern consistent with overall DEG dynamics, reaching its maximum at stage A4. The most significantly enriched pathways were primarily associated with carbon metabolism, amino acid metabolism, lipid metabolism, energy metabolism, signal transduction, and environmental adaptation. For instance, the number of DEGs involved in energy metabolism, lipid metabolism, transport and catabolism, signal transduction, and environmental adaptation reached 117 and 55, 92 and 65, 48 and 41, and 57 and 35 at days 4 and 6 of aging, respectively, highlighting extensive metabolic reorganization accompanying seed deterioration (Figure 4). These findings suggest that seed aging is associated with coordinated reprogramming across multiple metabolic and signaling pathways, potentially reflecting the gradual loss of physiological homeostasis and stress resilience during seed deterioration.

**Figure 5:**
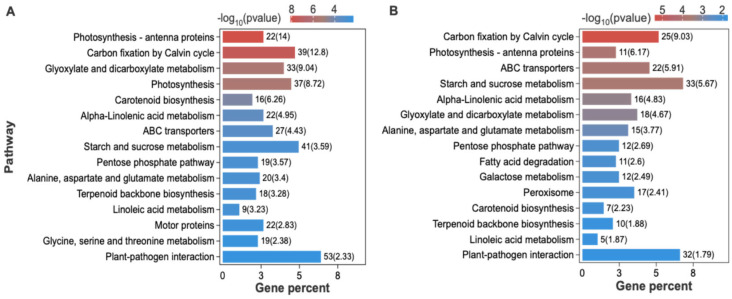
KEGG pathway enrichment analysis of DEGs under different aging durations. KEGG pathways significantly enriched in DEGs from the comparisons A4 vs. A0 (**A**) and A6 vs. A0 (**B**) are shown, highlighting the major biological processes affected by prolonged aging.

KEGG enrichment of the A0 vs. A4 and A0 vs. A6 comparisons revealed overlapping enrichment in “Photosynthesis-antenna proteins,” “Carbon fixation in photosynthetic organisms,” “Glyoxylate and dicarboxylate metabolism,” “ABC transporters,” “α-Linolenic acid metabolism,” and “Starch and sucrose metabolism.” In contrast, “Pyruvate metabolism,” “MAPK signaling pathway,” and “Peroxisome” were exclusively and significantly enriched in A6, suggesting their strong association with the terminal phase of seed viability loss. (Figure 5A,B).

**Figure 6:**
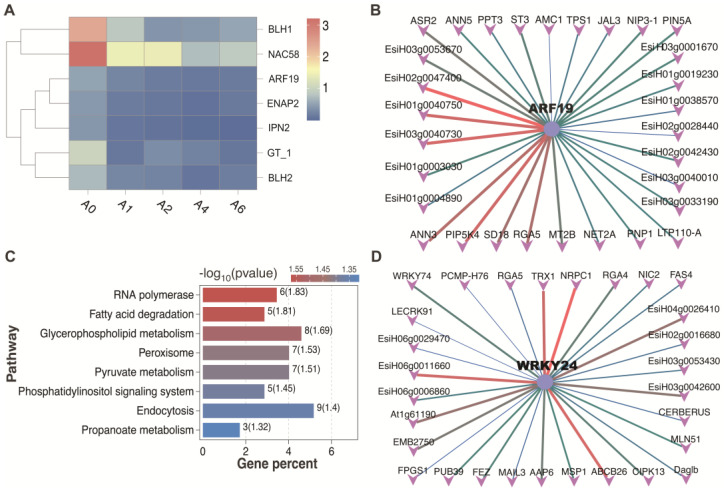
Transcription factor analysis of common and specific DEGs during seed aging in *E. sibiricus*. (**A**) Seven transcription factors commonly differentially expressed across all aging stages. (**B**) Regulatory network of *ARF19* and its predicted target genes. (**C**) KEGG enrichment analysis of 1136 specific DEGs identified in the A6 group. (**D**) Regulatory network of *WRKY24* and its predicted target genes. In the TF–target networks (**B**,**D**), line color indicates motif occurrence probability (red: higher; dark green: lower). Thicker lines indicate stronger predicted binding likelihood. All interactions are predicted and have not been experimentally validated.

Among the 220 core DEGs consistently altered across all time points—the majority were downregulated. These included heat shock proteins (*HSP18.9*, *HSP22.3*, and *HSP70*), sugar transporters (five *SWEET13* and one *SWEET3B*), and genes involved in hormone metabolism and transport (e.g., *PIN5A/PIN5B*, *ABCG14*, *CKX4*, *CYP734A4*, *ASR2*) (Figure 6A). Seven transcription factors were identified among the core DEGs, with *BLH1* and *NAC58* exhibiting notably high expression levels. Transcriptional network analysis of these 220 genes identified *ARF19* as a candidate central hub, predicted to control 30 downstream targets, including *ANN3*, *ANN5*, *PIP5K*, *RGA5* and *MT2B*, which are associated with ROS scavenging and stress responses (Figure 6B).

For the 1136 DEGs unique to A6, enrichment analysis revealed significant overrepresentation of pathways including “RNA polymerase”, “Fatty acid degradation”, “Glycerophospholipid metabolism”, “Peroxisome”, and “Pyruvate metabolism.” A separate network constructed from these genes identified *WRKY24* as a putative central regulator, predicted to target 29 genes, notably *TRX1*, *ABCB26* and *NRPC1*, which play roles in antioxidant defense, active transport and methylation regulation, respectively (Figure 6A,B).

### 2.4. Weighted Gene Co-Expression Network Analysis (WGCNA)

**Figure 7:**
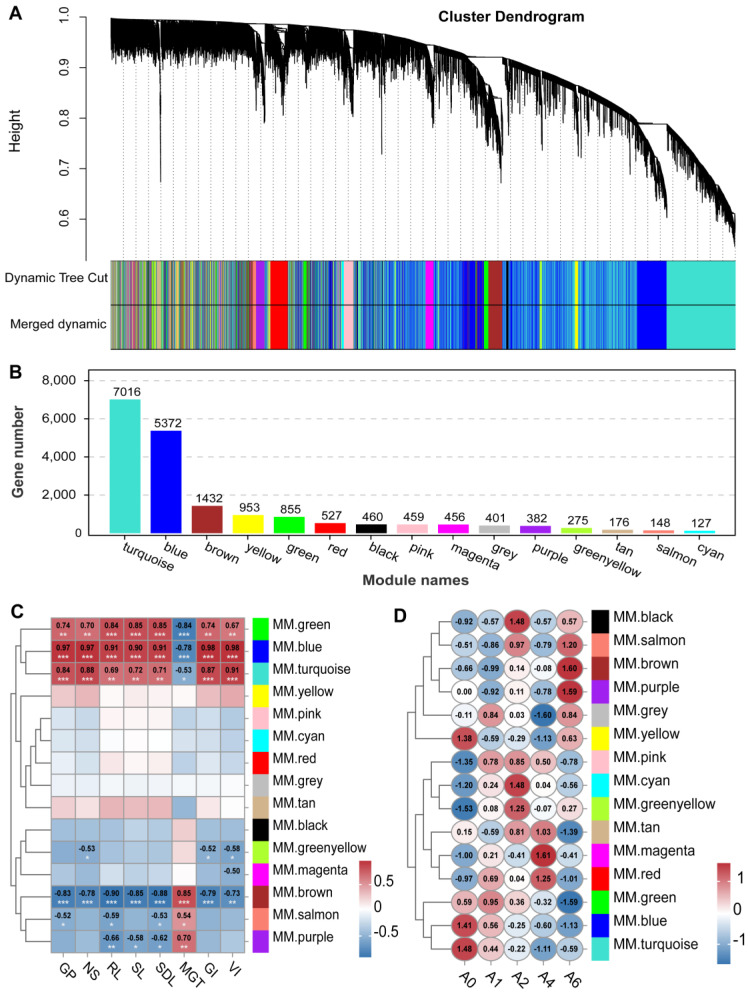
WGCNA of seed aging-related genes in *E. sibiricus*. (**A**) Gene dendrogram based on topological overlap dissimilarity. (**B**) Number of genes in each co-expression module. (**C**) Correlations between germination-related traits and modules (* *p* < 0.05; ** *p* < 0.01; *** *p* < 0.001). (**D**) Expression dynamics of representative modules across different aging stages.

WGCNA grouped all expressed genes into 15 distinct modules (Figure 7A). Among them, the turquoise and blue modules contained the largest number of genes (Figure 7B). Module–trait correlation analysis revealed seven modules that were significantly associated with seed vigor-related indices.

**Figure 8:**
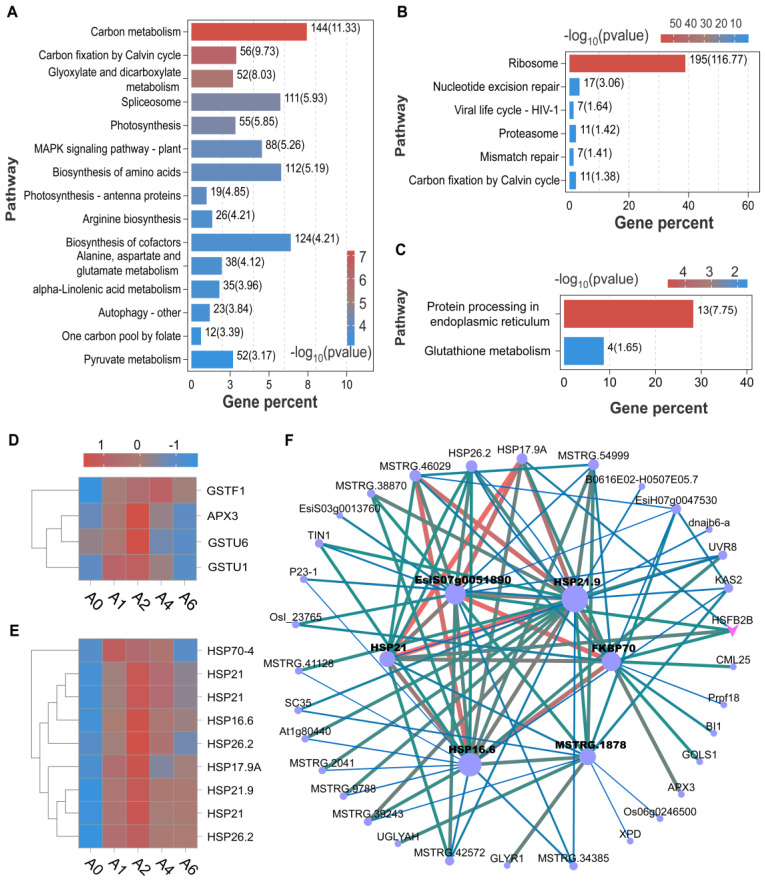
Functional enrichment and regulatory network analysis of key co-expression modules. (**A**–**C**) KEGG pathway enrichment of the turquoise (**A**), brown (**B**), and cyan (**C**) modules. (**D**,**E**) Heatmaps showing expression profiles of genes involved in GSH metabolism (**D**) and heat shock proteins (**E**) within the cyan module. (**F**) Regulatory network of the cyan module. In the network visualization, pink arrows represent transcription factors, circles denote genes, and lines indicate predicted interactions between genes or proteins. Node size reflects connectivity (larger nodes indicate higher connectivity), while edge thickness and color represent interaction strength (thicker and redder edges denote stronger associations). All interactions are predicted and have not been experimentally validated.

The turquoise, blue, and green modules exhibited progressive downregulation with aging, showing strong positive correlations with germination percentage (GP), radicle emergence percentage (EP), root length (RL), shoot length (SL), seedling length (SDL), germination index (GI), and vigor index (VI), but negative correlations with mean germination time (MGT) (Figure 7C). Functional enrichment of the turquoise and blue modules revealed strong associations with key pathways including carbon and lipid metabolism, RNA splicing, protein processing, amino acid biosynthesis, pyruvate metabolism, MAPK signaling, and cofactor synthesis (Figure 8A). Based on these associations, we hypothesize that the collective downregulation of these pathways may contribute to metabolic imbalance and loss of seed vigor. In contrast, the brown module displayed the opposite trend—its expression increased with aging and correlated negatively with vigor-related traits but positively with MGT (Figure 7C). It was primarily enriched for genes involved in ribosomal function and nucleic acid repair, suggesting a potential stress response mechanism (Figure 8B).

We also focused on several gene modules that exhibited transient upregulation followed by subsequent decline during the aging process, such as the pink, cyan, and green yellow modules (Figure 7D). KEGG enrichment analysis revealed that genes within these modules were primarily involved in protein processing in the endoplasmic reticulum, suggesting early activation of protein quality control mechanisms in response to aging-induced stress. Among them, the cyan module showed a more pronounced rise-and-fall pattern. This module was significantly enriched in the glutathione metabolism pathway, which is known to play a crucial role in maintaining redox homeostasis during seed aging. Key antioxidant enzyme genes within this pathway included *APX3*, *GSTF1*, *GSTU6*, and *GSTU1* (Figure 8D). Moreover, the cyan module contained several members of the HSP family, specifically including *HSP16.6*, *HSP17.9*, *HSP21*, *HSP21.9*, and *HSP70*. Among these, *HSP17.9*, *HSP21*, and *HSP70* were predicted to be regulated by the transcription factor HSFB2B and exhibited co-expression and potential functional interactions with genes involved in protein folding and stress response (Figure 8E,F).

**Figure 9:**
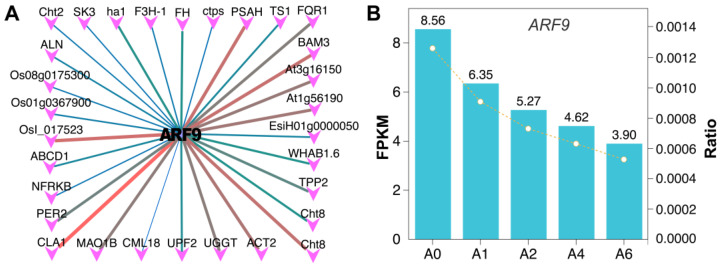
Transcriptional regulatory network analysis of DEGs significantly associated with seed vigor. (**A**) Interaction network of ARF9 and differentially expressed genes significantly correlated with seed vigor. The color of the connecting lines indicates motif occurrence probability, with red representing higher and dark green representing lower probabilities. The thickness of the lines reflects the likelihood of binding between transcription factors and target gene sequences (thicker lines indicate stronger binding potential). All interactions are predicted and have not been experimentally validated. (**B**) Expression trend of ARF9 during seed aging.

By integrating WGCNA modules with DEGs, we identified 384 high-confidence candidate genes strongly associated with seed vigor. Transcriptional network analysis of these genes revealed *ARF9* as a candidate central regulator predicted to target 30 downstream genes, including photosystem I subunit H (*PSAH*), flavodoxin-like quinone reductase 1 (*FQR1*), β-amylase 3 (*BAM3*), chitinase 8 (*CHT8*), UDP-glucose: glycoprotein glucosyltransferase (*UGGT*), cloroplastos alterados 1 (*CLA1*), monoamine oxidase B (*MAO1B*), and *PER2* (Figure 9A,B). These targets are involved in ROS metabolism, photosynthetic electron transfer, and cellular protein quality control, suggesting that *ARF9* may coordinate multiple physiological processes potentially crucial for seed survival.

### 2.5. Validation of RNA-Seq Data by RT-qPCR

To validate the reliability of the RNA-seq data, we performed RT-qPCR on seven candidate genes (*ARF9*, *ARF19*, *GSTF1*, *HSP70*, *APX3*, *HSFA2D*, and *WRKY24*) at the same aging time points used for RNA-seq. As shown in Figure S2A–G, each of the seven genes exhibited expression patterns consistent with the RNA-seq data across the aging time course. These results support the robustness of our RNA-seq-based findings.

## 3. Discussion

### 3.1. Key Metabolic Pathways Underlying Seed Vigor Decline

Seed aging is a multifactorial process characterized by cumulative physiological deterioration and global transcriptional reprogramming. The loss of seed vigor is closely associated with extensive metabolic alterations in key biochemical processes, including carbon, amino acid, and lipid metabolism [9,17]. In *E. sibiricus*, transcriptome profiling revealed widespread downregulation of genes associated with these fundamental metabolic pathways, as well as those involved in energy production and oxidative stress responses. Mitochondria, as the primary energy factories of plant cells, play a crucial role in determining whether seeds can successfully germinate. Aging often leads to mitochondrial structural abnormalities, impaired biogenesis, or reduced respiratory efficiency, which may contribute to insufficient energy supply and germination failure [12,18]. Moreover, mitochondria are the main sites of ROS generation in seeds. Excessive ROS accumulation during aging may further disrupt mitochondrial function, creating a vicious cycle that accelerates cellular damage and loss of viability [18]. At the later stages of aging, pathways such as pyruvate metabolism, MAPK signaling, and peroxisome function were particularly affected. This suggests that disruption of these processes may contribute to the terminal decline in seed vigor.

Pyruvate metabolism serves as a central metabolic hub linking glycolysis to the tricarboxylic acid (TCA) cycle. Disruption of this pathway is likely to reduce ATP synthesis and cause the accumulation of toxic intermediates such as acetaldehyde. Mitochondrial dysfunction during advanced aging may further impede pyruvate flux into the TCA cycle, thereby aggravating energy shortages and potentially accelerating vigor loss [19]. In closely related species such as oat and wheat (*Triticum aestivum*), pyruvate metabolism has been identified as a key pathway influencing seed aging [20,21]. Among the critical enzymes involved, acetyl-CoA synthetase (ACS) catalyzes the conversion of pyruvate to acetate, which is subsequently transformed into acetyl-CoA—an essential “metabolic switch” enabling entry into the TCA cycle. *ACS* has thus been recognized as a crucial gene in the regulation of seed aging [21]. Additionally, pyruvate kinase (PK), which catalyzes the terminal step of glycolysis, represents another pivotal enzyme in this pathway. Functional impairment of PK can block glycolytic flux, leading to glucose accumulation and a consequent decline in cellular energy levels. Such metabolic imbalance may further disturb the GA/ABA equilibrium, ultimately compromising seed vigor [22].

The MAPK signaling cascade functions as a pivotal transducer of both environmental and hormonal cues, mediating cellular responses to stress [23]. During seed aging, dysregulation of MAPK signaling can impair ROS detoxification and DNA or protein repair processes, thereby accelerating vigor decline [24]. Upon exposure to adverse environmental conditions or oxidative stress, the MAPK cascade is typically activated, leading to the phosphorylation of downstream transcription factors and enzymes that trigger the expression of antioxidant genes and regulate cell cycle progression and programmed cell death. This signaling pathway has been shown to be particularly sensitive to aging processes. In species such as oat and tomato (*Solanum lycopersicum*), MAPK-related genes exhibit pronounced alterations in expression during seed aging [10,24]. For example, in oat embryos imbibed for 24 h, the transcript levels of *WNK8* and *MAPK2* were significantly reduced in aged seeds compared with unaged controls [10], whereas an upregulation of *MPK3* and *MPK6* activity was observed during progressive aging in tomato [24]. Moreover, MAPK cascades can interact with hormonal signaling pathways to modulate seed vigor. For instance, the *OsMKKK62–OsMKK3–OsMPK7/14* module was reported to regulate ABA sensitivity and thus control seed dormancy and germination dynamics [25].

Similarly, peroxisomal dysfunction may exacerbate ROS accumulation during seed aging. Peroxisomes harbor CAT, a key antioxidant enzyme that decomposes H_2_O_2_, serving as a primary enzymatic defense against oxidative stress. Reduced CAT activity has been documented in several plant species during seed aging, often correlating with increased ROS levels and loss of vigor [10,24]. Excess hydrogen peroxide functions as a signaling molecule under normal conditions. However, when over-accumulated, it can act as a damaging agent, marking a critical biochemical signature of reduced seed viability [26,27].

### 3.2. Gene Co-Expression Modules Associated with Seed Vigor

Transcriptome-based modular analysis provides an efficient approach to dissect the complex molecular events underlying seed aging. In this study, several gene modules showed strong positive correlations with germination rate, seedling growth, vigor index, and germination index, and were progressively downregulated during aging. They were primarily enriched in pathways associated with carbon metabolism, lipid metabolism, RNA splicing, protein processing, amino acid biosynthesis, pyruvate metabolism, MAPK signaling, and cofactor biosynthesis. Together, these enrichments suggest a progressive decline in metabolic capacity and signal transduction efficiency during seed aging.

Conversely, a subset of pathways exhibited upregulation during aging, particularly at the early stages when seeds still retained partial germination capacity. These included genes associated with ribosomal function, protein processing, nucleic acid repair, and glutathione metabolism. The upregulation of genes related to ribosomal activity, protein folding, and DNA repair does not necessarily indicate true enhancement of these processes. Rather, it may reflect an emergency response to widespread macromolecular damage. Such activation likely represents an attempt by the seed to counteract or delay deterioration, although these repair mechanisms may be insufficient to offset the cumulative damage.

For instance, under aging-induced stress, cells often elevate the expression of molecular chaperones such as HSP70 and sHSP, which are activated by heat shock factors and are known to stabilize misfolded proteins and preserve seed vigor [28,29,30,31]. Although some of these heat shock proteins have been demonstrated to play protective roles during seed aging, severe oxidative stress can ultimately exceed their repair capacity. In addition, genes involved in glutathione metabolism were significantly enriched, highlighting its critical role in maintaining redox homeostasis and detoxification [10,32]. Enzymes participating in the AsA–GSH cycle, including DHAR, GR, and APX, have been extensively reported as key regulators of seed longevity [8,11,12]. Notably, the upregulated *APX* gene observed in this study encodes a key mechanism for hydrogen peroxide scavenging. It has been demonstrated that co-overexpression of *APX* and *CuSOD* can significantly enhance seed aging tolerance [11].

Both GSH-dependent and GSH-independent detoxification pathways contribute to maintaining seed vigor [10,33]. In rice, most genes associated with these pathways were significantly upregulated in aging-tolerant genotypes compared with aging-sensitive ones, and genes such as glyoxalase I (*GLYI3*) and Aldo-ketoreductase 1 (*AKR1*) have been experimentally validated to alleviate seed deterioration [34,35]. Furthermore, members of the phi and tau subfamilies of glutathione S-transferases (*GST*) were also upregulated during aging in *E. sibiricus*. Similar trends have been reported in sweet corn, where differences in ROS production and scavenging efficiency mediated by *GST* genes were identified as major determinants of seed aging tolerance [36]. Although phi and tau class *GSTs* are well recognized for their roles in abiotic stress responses, their precise functions during seed aging remain unclear and warrant further investigation. Taken together, the aforementioned HSPs and glutathione metabolism-related enzymes were co-localized within the same module. Based on these co-expression patterns, we hypothesize that both possess the potential to maintain seed vigor and may synergistically participate in the maintenance mechanism of seed viability during aging.

### 3.3. Transcriptional Regulators Orchestrating Seed Aging in E. sibiricus

Seed vigor is a complex quantitative trait governed by multiple genes and regulated by a diverse array of transcription factors, including members of the bZIP [9], bHLH [37], MYB [38], NAC [39], and WRKY families [40]. These factors integrate signals from endogenous hormones, redox homeostasis, and environmental stresses to coordinate the maintenance of seed viability. Integrating these findings with prior studies, our co-expression analysis identified several transcription factors as candidate regulators of seed aging in *E. sibiricus*. Among these, WRKY24, ARF9, and ARF19 emerged as high-confidence candidate regulators associated with seed viability decline.

The downregulation of *WRKY24* in aged seeds, coupled with its strong association with genes involved in oxidative stress mitigation (*TRX1*), protein synthesis (*NRPC1*), and transport (*ABCB*), suggests that *WRKY24* may participate in the coordination of these processes. However, whether WRKY24 directly regulates these targets remains to be determined. Comparative analysis reveals that WRKY-mediated regulatory mechanisms may vary across species. For instance, different WRKY members in rice [9] and alfalfa [40] exhibit diverse, and sometimes contrasting, expression patterns during aging. This variation highlights the complexity of the WRKY regulatory network and suggests that the function of *WRKY24* in *E. sibiricus* might be species- or condition-specific.

Similarly, the auxin signaling pathway emerged as a significant regulatory hub in our study. The identification of *ARF9* and *ARF19* aligns with the established role of auxin in seed longevity across species such as rice [41], Arabidopsis [42], and maize [43]. Notably, the predicted targets of these ARFs in our network—including *ANN5* and *MT2B* for ARF19, and *FQR1*, *PER2*, and *MAO1B* for ARF9—are associated with redox homeostasis. Given that members of the peroxidase (PER) families are known to influence seed storability, our findings raise the possibility that ARF9 and ARF19 may contribute to seed vigor, at least in part, by regulating genes involved in redox homeostasis.

Based on the above observations, we propose a working hypothesis for future investigation: the transcription factors WRKY24, ARF9, and ARF19 may represent key nodes in a regulatory network governing seed longevity in *E. sibiricus*. Specifically, WRKY24 might influence oxidative stress responses, while the ARFs could mediate redox balance through their respective target genes. Whether these pathways function independently or exhibit crosstalk (e.g., via shared target genes or regulatory feedback) remains an open question. Functional validation through genetic manipulation (e.g., knockout or overexpression) and molecular assays (e.g., ChIP-qPCR, EMSA) will be essential to elucidate the precise mechanisms by which these transcription factors regulate seed aging.

In the present study, transcriptomic profiling combined with WGCNA provided valuable insights into the regulatory networks underlying seed aging in *E. sibiricus*. This work represents a transcriptome-wide characterization of seed aging in a perennial alpine grass species and one of the few studies focusing on perennial forage grasses. Unlike previous studies in annual crops such as rice, oat, wheat, and maize, which have focused on short-lived species, our findings in *E. sibiricus* reveal candidate regulators (e.g., *WRKY24*, *ARF9*, *ARF19*) and co-expression modules (e.g., linking heat shock proteins with glutathione metabolism) that have not been previously reported in perennial grasses adapted to high-altitude environments. These insights extend beyond annual crop models by highlighting potential mechanisms that may underlie seed longevity in ecologically and agronomically important perennial species.

However, several limitations should be acknowledged. First, although accelerated aging (45 °C, 80% RH) is a widely accepted and practical experimental system for studying seed aging, it remains an artificial proxy for natural aging during long-term storage. The transcriptomic responses observed under these conditions may not fully capture all physiological and molecular events occurring under conventional storage conditions. Second, the present study focuses primarily on transcript-level changes, whereas seed aging is also strongly influenced by post-transcriptional regulation, protein turnover, enzyme activity, metabolite accumulation, and membrane damage. Therefore, our conclusions are largely correlative and hypothesis-generating. In addition to the absence of functional validation (e.g., gene overexpression or silencing), these limitations call for cautious interpretation of the proposed regulatory framework. Future studies integrating transcriptomics with proteomics, metabolomics, ROS measurements, and antioxidant enzyme assays will be necessary to build a more complete mechanistic model of seed aging in *E. sibiricus*. In addition, functional characterization of key hub genes (e.g., *WRKY24*, *ARF9*, *ARF19*) via virus-induced gene silencing (VIGS) or heterologous expression will be required to substantiate the proposed regulatory framework.

## 4. Materials and Methods

### 4.1. Plant Material and Artificial Aging Treatment

Seeds of *E. sibiricus* L. cultivar ‘Maiwa’ were collected in August 2023 from the experimental station of the Sichuan Academy of Grassland Sciences, located in Hongyuan County, Sichuan Province, China. After harvest, seeds were carefully cleaned to remove empty or shriveled kernels. According to the International Seed Testing Association (ISTA) protocols, seed moisture content was adjusted to 10% prior to aging treatments [10]. The initial standard germination rate of the seed lot was 94%.

Seeds with a moisture content of 10% were equilibrated at 20 °C for 3 days prior to aging treatment. After equilibration, seeds were evenly distributed in sterile plastic Petri dishes (13.5 cm × 13.5 cm) without overlapping, with 200 seeds per dish. The dishes were then placed in a constant temperature and humidity seed aging chamber (HWS-160, Ningbo Jiangnan Instrument Factory, Ningbo, China) set to 45 °C and 80% relative humidity (RH) for artificial accelerated aging [9]. Based on preliminary germination tests (Figure S3), seeds were collected after 1, 2, 4, and 6 days of accelerated aging. For each time point, three independent biological replicates were prepared, each consisting of 200 seeds. After aging, all samples were equilibrated at room temperature for 24 h and designated as A1, A2, A4, and A6, respectively. Untreated control seeds (A0) were kept under the same conditions without aging and reserved for subsequent analyses.

### 4.2. Germination Assay and Vigor Assessment

Seed germination percentage was evaluated based on the count of normal seedlings as per ISTA (2019) guidelines. Each treatment consisted of three biological replicates, with each replicate comprising 50 seeds. Seeds were placed on sterile plastic Petri dishes (12 cm × 12 cm) and incubated in a growth chamber at a constant 25 °C under a photoperiod of 8 h light/16 h darkness and a light intensity of 1200 lux.

The number of seeds with a radicle emergence exceeding 2 mm was recorded daily. From these data, the mean germination time (MGT) was calculated, and germination kinetics curves were plotted. On the 12th day of the germination assay, final assessments were conducted, including germination percentage (GP, percentage of normal seedlings) and emergence percentage (EP, percentage of radicle emerged seeds), as well as measurements of shoot length (SL), root length (RL), seedling length (SDL), germination index (GI), and vigor index (VI).

MGT was calculated according to the following formula: MGT = ∑(N×T)/N

GI was calculated according to the following formula: GI = ∑(Gt/Dt)

VI was calculated according to the following formula: VI = GI×SL

Where “T” is the number of days counted from the beginning of radicle emergence exceeding 2 mm, “N” is the number of seeds emerged on day T. “Gt” is the number seeds emerged a time “t” days and “Dt” indicates the corresponding number of seed emergence days.

### 4.3. Transcriptome Sequencing and Analysis

#### 4.3.1. RNA Extraction, cDNA Library Construction, and Sequencing

Seed samples from each aging treatment were rapidly and thoroughly ground in liquid nitrogen. For each aging time point (A0, A1, A2, A4, A6), three independent biological replicates were prepared. Total RNA was extracted from 0.1 g of the resulting powder per replicate. To ensure high-quality sequencing data, the integrity, purity, and concentration of the RNA were rigorously assessed. Ribosomal RNA was depleted using a commercial kit to enrich messenger RNA (mRNA).

The purified mRNA was enriched using mRNA Capture Beads, fragmented by exposure to elevated temperature, and then used as a template for first-strand cDNA synthesis in a reverse transcription master mix. Second-strand cDNA synthesis was subsequently performed, followed by end repair and adenylation. The resulting cDNA fragments were purified and size-selected using Hieff NGS^®^ DNA Selection Beads, amplified via PCR to construct the final sequencing libraries, and subjected to quality control. Paired-end (PE150) sequencing was performed on an Illumina NovaSeq X plus (Illumina, Inc., San Diego, CA, USA), generating approximately average raw data volume ~6.7 Gb per sample.

Raw sequencing reads were processed using fastp [44] to obtain high-quality clean reads. The clean reads had an average Q30 score > 93%. These clean reads were then aligned to the *E. sibiricus* reference genome using the HISAT2 software (v2.1.0) [45]. The average mapping rate across all samples was approximately 81% (see Table S1 for detailed quality metrics per sample). Based on the alignment results from HISAT2, transcriptomes were assembled and quantified for each sample using StringTie [46], and gene expression levels were estimated as read counts using RSEM [47].

#### 4.3.2. Differential Gene Expression and Functional Enrichment Analysis

Differential gene expression analysis was performed using the DESeq2 software package (http://www.bioconductor.org/packages/release/bioc/html/DESeq2.html, accessed on 20 June 2025) in R, which utilized the raw read count data generated from the expression level quantification. The analysis involved data normalization, statistical hypothesis testing based on a negative binomial distribution model, and multiple-testing correction to generate False Discovery Rate (FDR) values. Significant DEGs were identified using DESeq2 with an FDR-adjusted *p*-value < 0.05 and |log2(Fold Change)| > 2.

Gene Ontology (GO, https://geneontology.org/, accessed on 25 June 2025) term enrichment analysis for the identified DEGs was conducted by mapping the genes to the GO database. The number of DEGs associated with each GO term was calculated to generate a list of enriched functional categories. Furthermore, Kyoto Encyclopedia of Genes and Genomes (KEGG, https://www.genome.jp/kegg/, accessed on 25 June 2025) pathway enrichment analysis was employed to identify the most significantly overrepresented biochemical metabolic and signal transduction pathways associated with the DEGs.

#### 4.3.3. Weighted Gene Co-Expression Network Analysis (WGCNA) and Transcription Factor Target Prediction Analysis

A weighted gene co-expression network was constructed using the WGCNA package v 1.73 (https://cran.r-project.org/web/packages/WGCNA/index.html, accessed on 20 July 2025) in R. Prior to analysis, genes with low expression levels (counts < 5 across samples) were filtered out. The resulting gene expression matrix was then used to construct co-expression modules with the following parameters: a soft-thresholding power of 8, an unsigned Topological Overlap Matrix type, a merge cut height of 0.25, and a minimum module size of 50. This process clustered genes into 15 distinct co-expression modules.

For in silico prediction of transcriptional targets, position weight matrices representing the DNA-binding motifs of TFs were retrieved from the JASPAR database (https://jaspar.elixir.no/, accessed on 5 July 2025). These motifs were subsequently used to scan the promoter sequences of genes using the FIMO software suite within the MEME package (https://web.mit.edu/meme/current/share/doc/fimo.html, accessed on 5 July 2025), identifying statistically significant putative binding sites. Selected TF-target gene pairs can be visually represented through an interactive online tool that generates a regulatory network diagram, illustrating the potential associations between transcription factors and their putative target genes.

#### 4.3.4. Real-Time qPCR (RT-qPCR) Analysis

Total RNA was extracted from seed samples using the Quick RNA Isolation Kit (Huayueyang Biotech Co., Ltd., Beijing, China) following the manufacturer’s instructions. First-strand cDNA was synthesized from 1 μg of total RNA using the EasyScript^®^ All-in-One First-Strand cDNA Synthesis SuperMix for qPCR Kit (TransGen Biotech Co., Ltd., Beijing, China). RT-qPCR was carried out on an Archimed R4 qPCR System using the One Step SYBR PrimeScript RT-PCR Kit II (TaKaRa, Dalian, China). The amplification conditions were as follows: an initial denaturation at 95 °C for 3 min, followed by 40 cycles of 95 °C for 10 s and 60 °C for 30 s. The *EIF4A* gene was used as an internal reference for normalization of gene expression levels. Relative transcript abundance was calculated using the 2^–ΔΔCt^ method. Gene-specific primers were designed using Primer Premier 5.0 software (Premier Biosoft International, Palo Alto, CA, USA) and are listed in Table S2. All reactions were performed in three independent biological replicates.

#### 4.3.5. Statistical Analyses

All statistical analyses were conducted using one-way ANOVA followed by Duncan’s multiple range test (*p* < 0.05) in SPSS Statistics version 22.0 (SPSS Inc.; Chicago, IL, USA). Graphs illustrating seed germination parameters, gene expression and correlation analysis were plotted with GraphPad Prism version 9 (GraphPad Software Inc., San Diego, CA, USA). All the visualizations (such as heatmaps, and network maps) were made by an online website (https://www.omicsmart.com/, accessed on 8 August 2025).

## 5. Conclusions

This study provides a comprehensive transcriptomic analysis of seed aging in *E. sibiricus*, revealing dynamic transcriptional reprogramming and key regulatory pathways associated with seed vigor decline. Artificial aging led to a progressive loss of germination capacity, accompanied by the global downregulation of genes involved in metabolism, stress signaling, and redox homeostasis. Integrative analyses identified that pyruvate metabolism, the MAPK signaling pathway, and peroxisome function are strongly associated with seed vigor decline during the late stages of aging. Furthermore, co-expression network analysis revealed that genes encoding heat shock proteins and glutathione metabolism-related enzymes were co-localized within the same module, suggesting a possible synergistic role in preserving seed viability during aging. In addition, WRKY24, ARF9, and ARF19 were identified as potential core transcriptional regulators. Among these, *WRKY24* may participate in the aging process by potentially modulating antioxidant defense-related genes (e.g., *TRX1*), while *ARF9* and *ARF19* may influence ROS metabolism through their predicted downstream target genes, including *FQR1*, *PER2*, *MAO1B*, *ANN5*, and *MT2B*. In summary, this study contributes to the understanding of the molecular mechanisms of seed vigor deterioration in perennial grasses and offers potential candidate gene resources for improving seed storability and supporting germplasm conservation in *Elymus* and related species.

## Data Availability

The original contributions presented in the study are included in the article/Supplementary Materials. Raw sequencing data of the transcriptome used in the current study are available in the NCBI’s Sequence Read Archive (SRA, https://www.ncbi.nlm.nih.gov/sra, accessed on 21 October 2025) under the BioProject PRJNA1347986.

## Supplementary Materials

The following supporting information can be downloaded at https://www.mdpi.com/article/10.3390/plants15091328/s1, Table S1: Summary of RNA-seq data quality and mapping statistics; Table S2: Primer sequences of seven selected genes for qRT-PCR validation; Figure S1: Principal component analysis (PCA) of transcriptome samples across aging time points; Figure S2: RT-qPCR validation of seven selected gene expression patterns during seed aging in *E. sibiricus*; Figure S3. Germination of *E. sibiricus* seeds in preliminary aging tests.
