# Supplementary material for: Transcriptomic Profiling Reveals the Seed Aging Process in Elymus sibiricus, a Dominant Alpine Grass
Source: Plants (Basel). 2026 Apr 27;15(9):1328. doi: 10.3390/plants15091328 (PMC13165205; doi:10.3390/plants15091328)
Supplement: Supplementary file 1 [file plants-15-01328-s001.zip › plants-4236059-supplementary.pdf]

Table S1. Summary of RNA‑seq data quality and mapping statistics.

Sample	RawData(bp)	CleanData(bp)	Q30(%)	Total Mapped(%)
A0-1	6706114800	6677334188	6260572843 (93.76%)	78.02%
A0-2	6641020800	6610548275	6139744738 (92.88%)	77.31%
A0-3	7009570800	6983154205	6528960627 (93.50%)	78.95%
A1-1	6603589500	6574940264	6137873307 (93.35%)	80.59%
A1-2	5988283800	5965948882	5552165088 (93.06%)	79.09%
A1-3	6421715100	6396880288	5972171290 (93.36%)	78.08%
A2-1	7476379200	7447572676	6942598454 (93.22%)	80.92%
A2-2	5899383000	5878539923	5483881700 (93.29%)	80.99%
A2-3	6865021200	6837334533	6377124766 (93.27%)	81.50%
A4-1	6246029700	6218195464	5802355202 (93.31%)	82.84%
A4-2	6226443900	6200020185	5773348324 (93.12%)	83.56%
A4-3	6048697500	6022617775	5599258463 (92.97%)	83.76%
A6-1	6464874900	6439662570	6000035735 (93.17%)	82.18%
A6-2	8133970500	8102367343	7563260766 (93.35%)	81.90%
A6-3	7819078200	7789314534	7272992073 (93.37%)	82.29%
Mean	6703344860	6676295407	6227089558 (93.27%)	80.90%

Table S2. Primer sequences of seven selected genes for qRT-PCR validation.

Gene ID	Gene Name	Forward Primer (5' - 3')	Reverse Primer (5' - 3')
EsiH02g0027330	*ARF9*	GAGCTATGGCATGCTTGTGC	AAAGACACTCCAGCCAGTCG
EsiH07g0015040	*ARF19*	ATGTTCGCCGATGTCGTCTA	CATGTTGCTGTCGTGGTCTT
EsiH01g0039070	*GSTF1*	GTGTACGAGTGCCTCATCAAC	GTAGACCTCCAGCACCTTCTT
EsiS05g0049000	*HSP70*	ACGGAGAAGCAACTGAAGGA	TGTGTCGATCCATCAGCAATG
EsiS02g0055560	*APX3*	CAACGAGAACTACCGCTGTC	AACCACTGAGCCTCCTCTTG
EsiH04g0011220	*HSFA2D*	AGGAGGAGGAGGAGTTGCT	CACCAGGTCGAACGTCTTG
EsiH01g0016140	*WRKY24*	CCCCTCGCATCAGTTCAACT	GCCTGCTCCACCTTCTTCTT

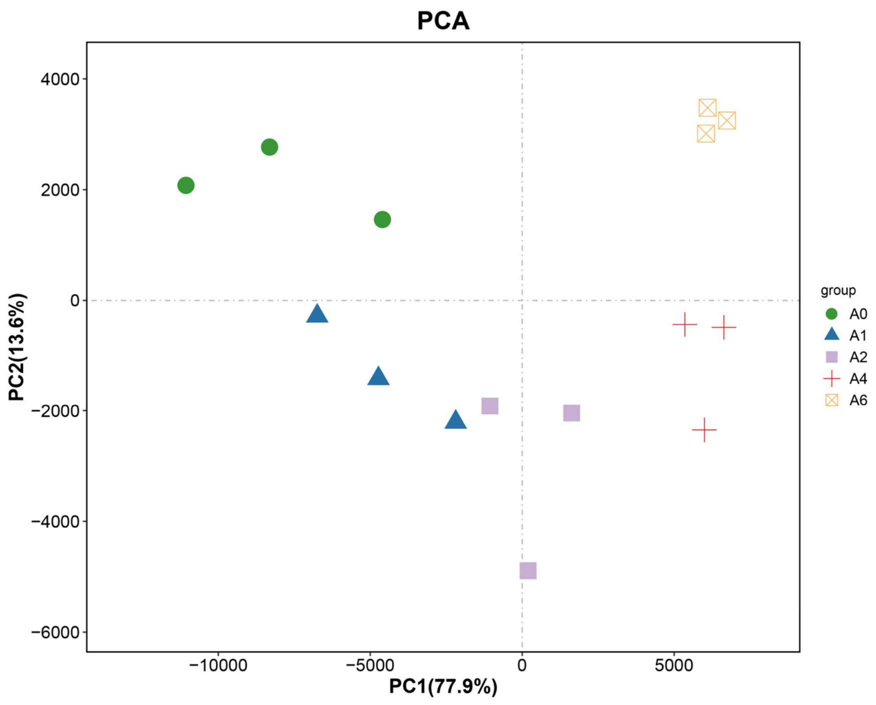

Figure S1. Principal component analysis (PCA) of transcriptome samples across aging time points.

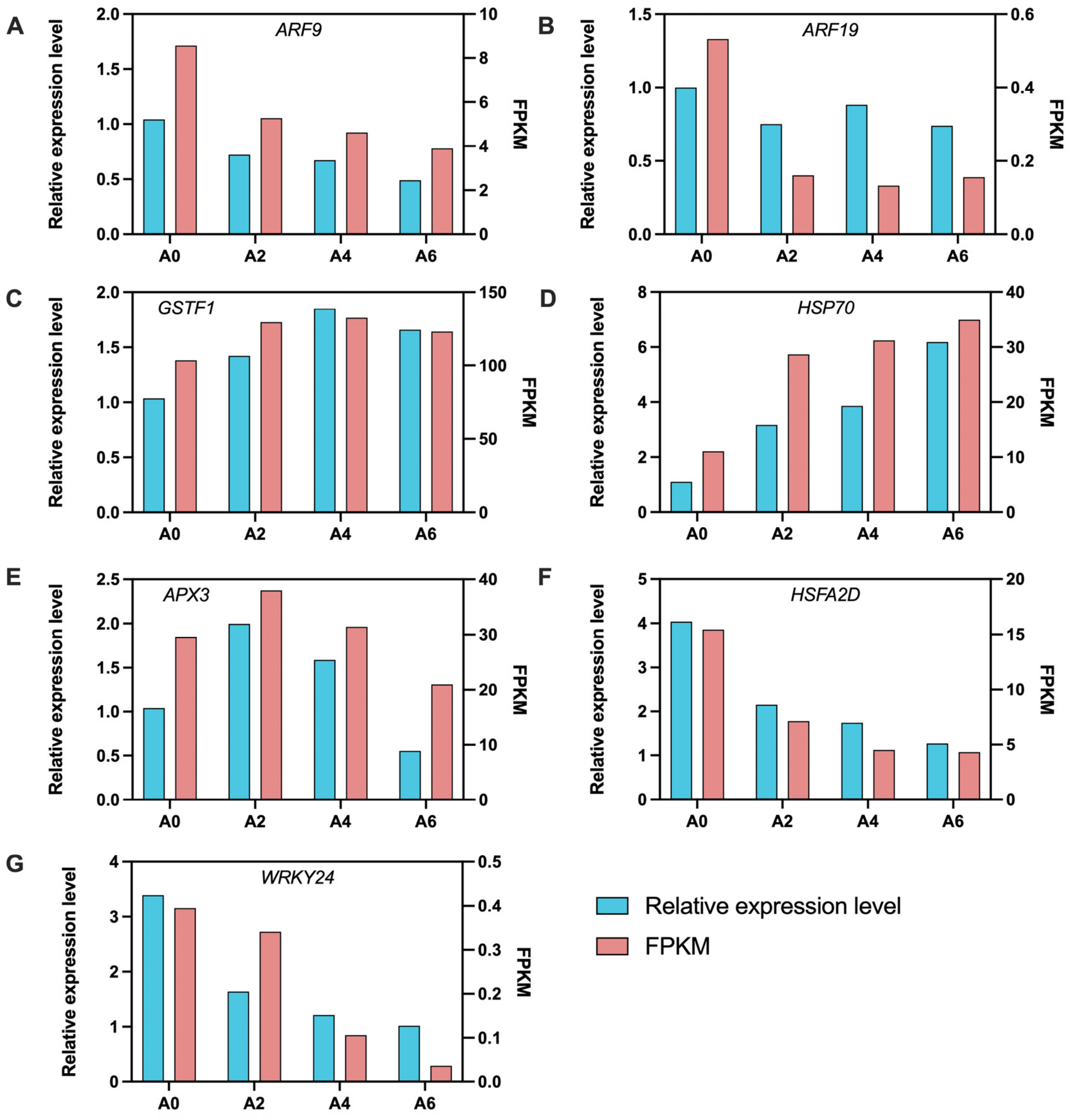

Figure S2. RT-qPCR validation of seven selected gene expression patterns during seed aging in *E. sibiricus*.

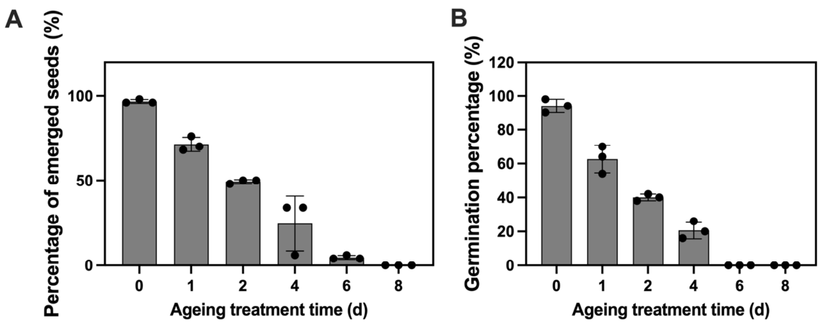

Figure S3. Germination of *E. sibiricus* seeds in preliminary aging tests.
